# Diffusion Kurtosis Imaging of Microstructural Alterations in the Brains of Paediatric Patients with Congenital Sensorineural Hearing Loss

**DOI:** 10.1038/s41598-017-01263-9

**Published:** 2017-05-08

**Authors:** Wenbin Zheng, Chunxiao Wu, Lexing Huang, Renhua Wu

**Affiliations:** 1 Department of Radiology, the Second Affiliated Hospital, Medical College of Shantou University, Shantou, 515041 China; 2 China Provincial Key Laboratory of Medical Molecular Imaging, Guangdong, Shantou 515041 China

## Abstract

Our aim was to assess microstructural alterations in the cerebrums of paediatric patients with congenital sensorineural hearing loss (SNHL) using diffusion kurtosis imaging (DKI). Seventy-two paediatric SNHL patients and 38 age-matched healthy volunteers were examined via DKI using a 3.0 T magnetic resonance (MR) imager. Fractional anisotropy (FA) and mean kurtosis (MK) values were computed for 12 cerebral regions in both the controls and the SNHL patients. Compared with patients below age 3, patients in the older age group were found to have more significant differences in MK than in FA, and these appeared in more major areas of the brain. In contrast, in 1- to 3-year-old children, a few major brain areas exhibited differences in FA, but none exhibited appreciable differences in MK. There were significant decreases in the FA or MK values (*P* < 0.05, *all*) in more areas of the brain in patients with lesions than in patients with normal-appearing brains. DKI offers comprehensive measurements for quantitative evaluation of age-related microstructural changes in both white and grey matter in SNHL patients. DKI scans of children with SNHL exhibiting significant decreases in MK might play an important role in evaluating the severity of developmental delay.

## Introduction

To date, the earlier identification of childhood deafness has led to greater opportunities for early intervention. Advances in cochlear implantation (CI) technology have yielded an effective treatment strategy that can restore hearing in children affected by sensorineural hearing loss (SNHL); this treatment can enable normal speech and language development. There is now abundant evidence that early CI in a child with SNHL is advantageous^1–6^.

Although imaging provides valuable preoperative information about the inner ear, the vestibulocochlear nerve, and the brain in the evaluation of children with congenital SNHL who are candidates for CI surgery, the large variability in outcome indicates that there are still CI-recipient children who lag behind other CI-recipient children in terms of recovery of auditory and language functions. Thus, it is likely that some CI-recipient children have deficits in both the auditory and cognitive areas that underlie the development of language. Preoperative magnetic resonance imaging (MRI) scans of the brain are routinely performed before CI, not only to evaluate the status of the inner ear and cochlear nerve but also to screen for central nervous system (CNS) disorders^7^. However, the relationships between the severity of a child’s clinical presentation and the severity of the CNS abnormalities observed on the MRI scan are not fully understood.

Previous diffusion tensor imaging (DTI) studies demonstrating changes in fractional anisotropy (FA) in children with SNHL have revealed that such children have a developmental delay in the myelination of the auditory neural pathway, thus suggesting that early CI might be more effective than later CI in restoring hearing in children with SNHL^8–10^.

Recently, diffusion-weighted techniques that exploit diffusional non-Gaussianity have been developed, and diffusion kurtosis imaging (DKI), which measures both the Gaussian and non-Gaussian properties of water diffusion, provides sensitive and comprehensive measurements for the quantitative evaluation of age-related microstructural changes. The mean kurtosis (MK), as an imaging marker, has been demonstrated to be sensitive to structural changes in both anisotropic tissue, such as white matter (WM), and isotropic tissue, such as grey matter (GM), thus affording information about tissue microarchitecture that is complementary to measures such as the FA and mean diffusivity (MD)^11–16^. DKI is therefore likely to be a more valuable technique than DTI for studying the developing brain. DKI has also been applied to various pathological states, including Alzheimer’s disease, schizophrenia, stroke, cerebral gliomas, multiple sclerosis, and attention deficit/hyperactivity disorder^17–23^.

In this study, we used DKI as a novel imaging tool to study children with SNHL and estimate differences in the microstructural integrity of the auditory pathway, auditory cortex, language-related cortex, and learning and memory function–related cortex during the first 7 years of life. We sought to determine whether DKI could detect preoperative microstructural changes in the white and grey matter of children who were to receive CIs and whether it could provide helpful additional information about the indication and selection of patients to receive a cochlear implant.

## Results

### Conventional MRI findings

**Figure 1:**
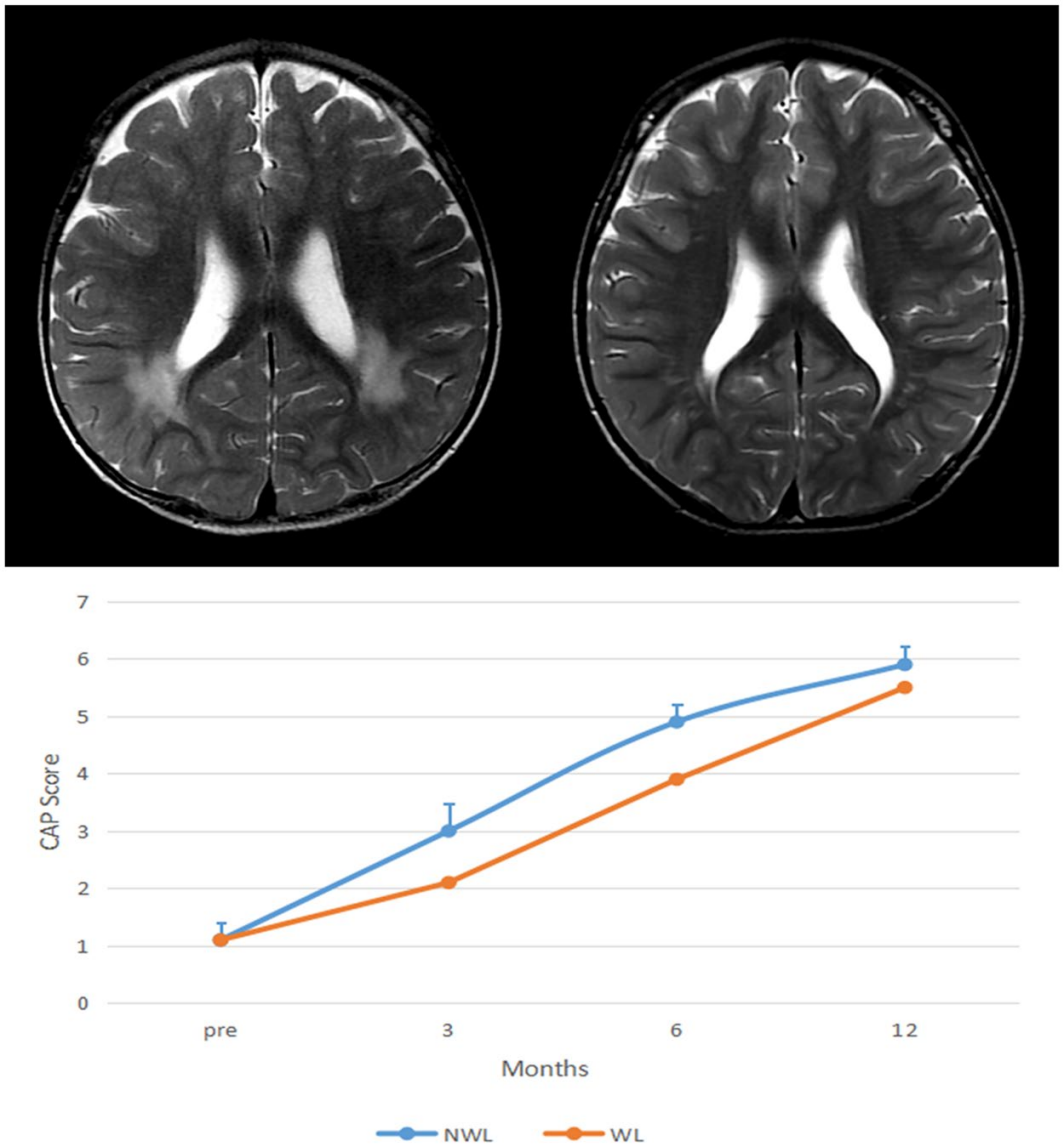
Brain MRI of a boy with SNHL and his CAP score change after CI. (**a**) At 15 months of age, with multiple and diffuse increased white matter T2 signals in the periventricular regions. (**b**) At 4 years of age, only focal high-signal lesions are seen in the periventricular white matter. (**c**) After implantation, the boy’s CAP score steadily improved and reached its highest level after 12 months, equalling that of the group without brain lesions; however, at 3 and 6 months after CI, he had a lower score than that of the group without brain lesions.

Using conventional MRI, no anatomical abnormalities of the inner ear and no cochlear nerve deficiencies were found in any of our subjects; however, brain lesions in the white matter (WL) (focal parenchymal high signal lesions in the periventricular white matter (focal parenchymal lesions)) and multiple diffuse, deep, white matter, high-signal lesions (diffuse parenchymal lesions) were observed in 39 of our 72 subjects. Non-white matter lesion (NWL) were observed in 33 of our 72 subjects. No signal changes were noted in the grey matter. Table 1 indicates no significant difference between the 2 age groups in terms of the proportions of patients with abnormalities detected in whole-brain images. Among 20 children who had received CIs, we found that 6 had brain lesions in the white matter according to conventional MRI. Figure 1 shows the abnormal brain MRIs of a boy with SNHL at 15 months of age (a). At 4 years of age, the abnormalities in high signal lesions of the white matter had almost disappeared (b). After implantation, the boy’s categories of auditory performance (CAP) score steadily improved and reached its highest level after 12 months, equalling that of the group without brain lesions; however, at 3 and 6 months after CI, he had a lower score than did the group without brain lesions (c).

**Table 1:** Proportions of Patients with Abnormalities Detected in the Whole-Brain Images in the 2 Age Groups of Patients with SNHL*.

SNHL Patients	Normality of Whole Brain Imaging	Abnormality on Whole Brain Imaging	Total
>3-year-olds	21 (52.5%)	19 (47.5%)	40 (55.5%)
1 to 3-year-olds	14 (43.7%)	18 (56.3%)	32 (44.5%)
Total	35 (48.6%)	37 (51.4%)	72 (100%)

*Pearson’s chi-square test *P* = 0.308.

### DKI

**Figure 2:**
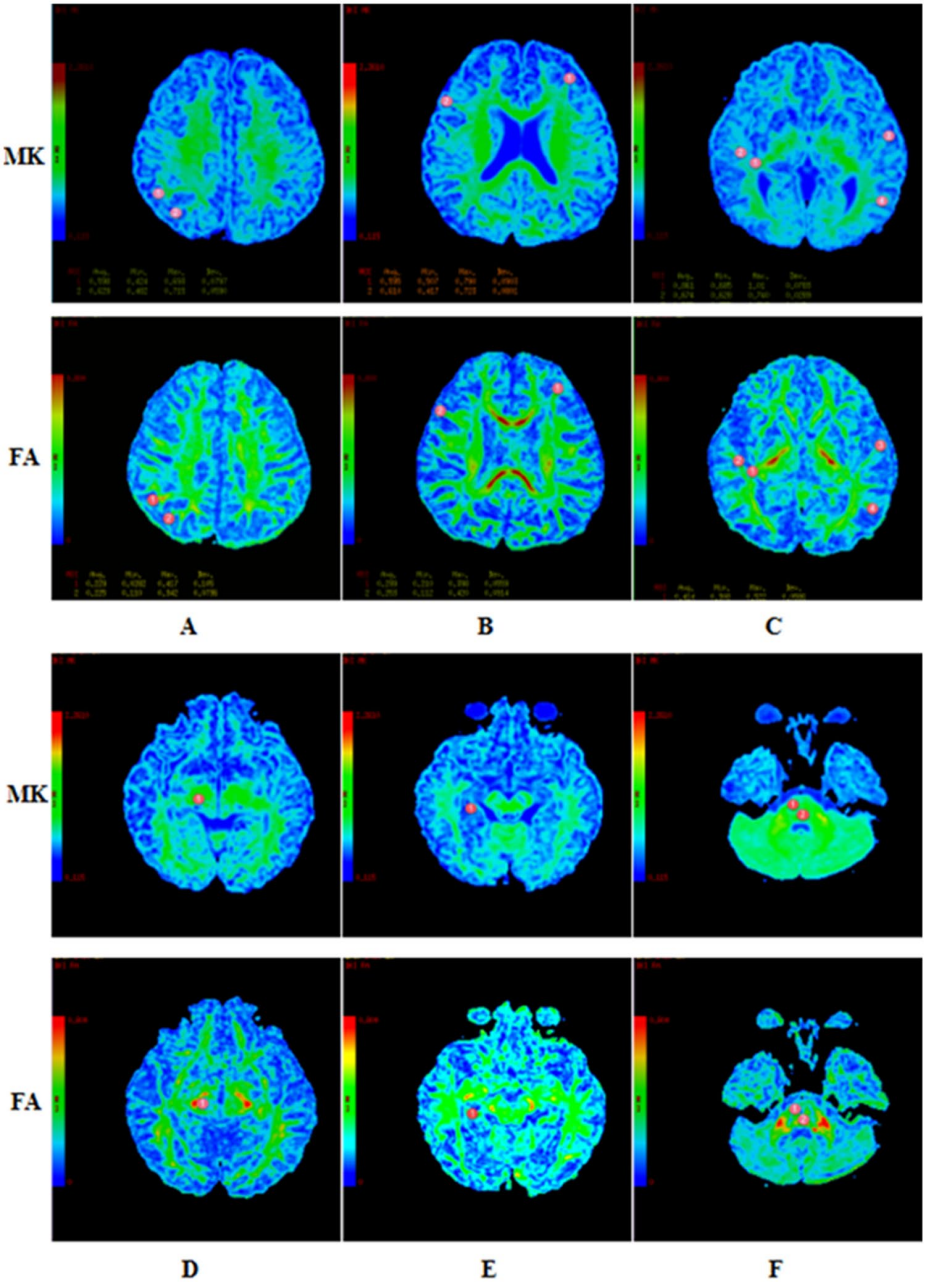
Representative DKI of the ROIs. The red circle indicates the selected ROI. (**A**) ① angular gyrus ② supramarginal gyrus. (**B**) ① middle frontal gyri ② inferfrontal gyri. (**C**) ① auditory radiation ② white matter of Heschl’s gyrus ③ middle temporal gyri ④ superior temporal gyri. (**D**) ① medial geniculate body. (**E**) ① hippocampus. F ① trapezoid body ② superior olivary nucleus.

**Figure 3:**
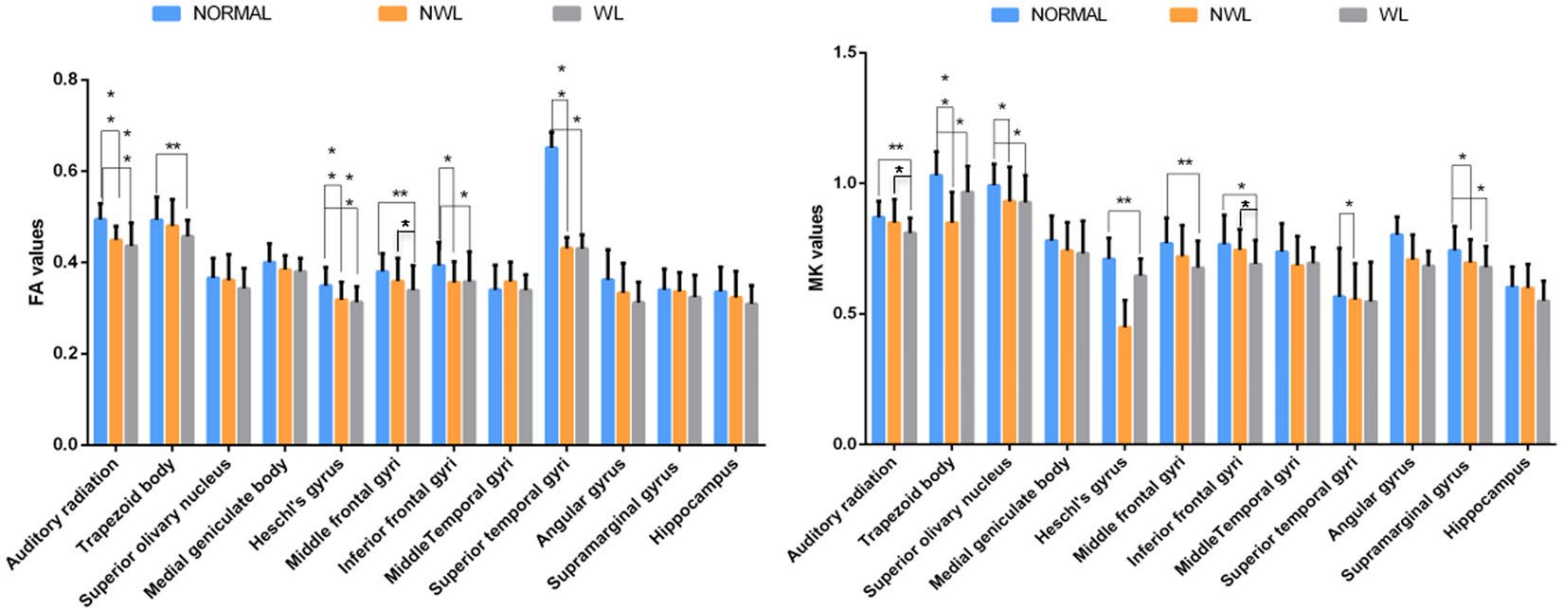
Significant differences in the FA (left) and MK (right) values in the various brain regions of controls and patients (NML or WL) above 3 years of age (*P < 0.05, **P < 0.01).

**Figure 4:**
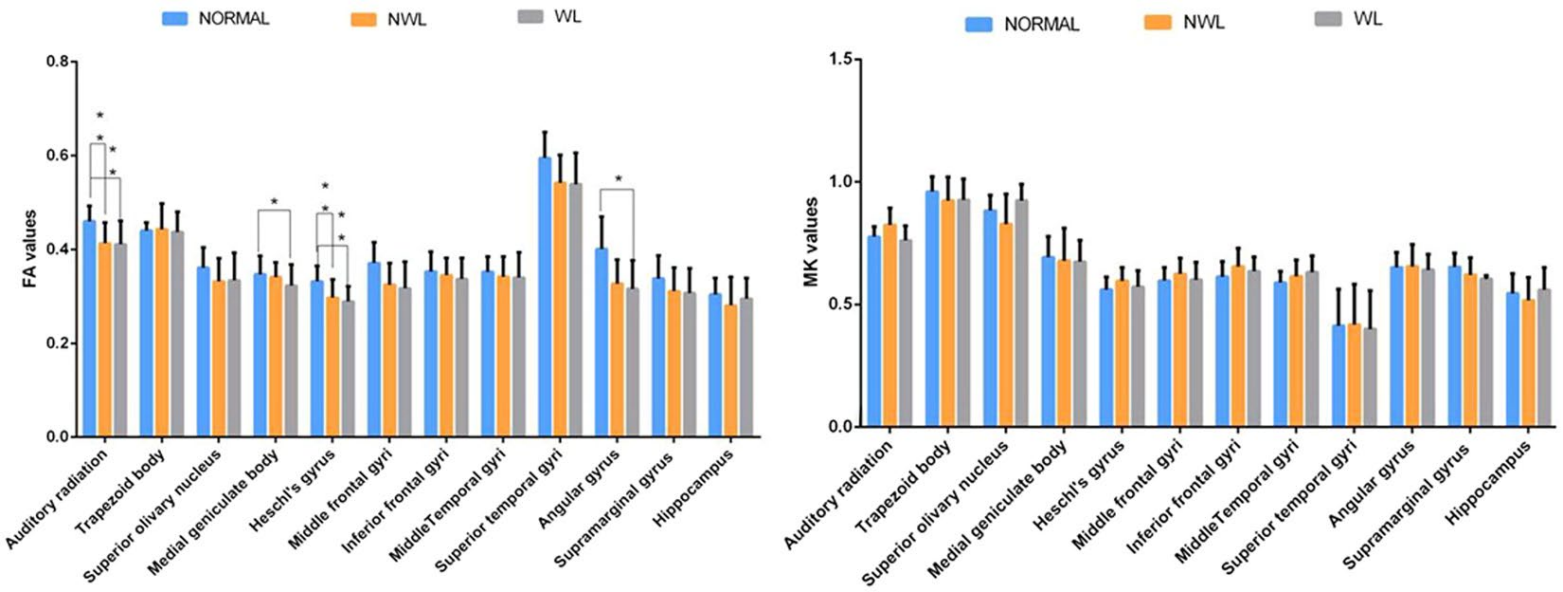
Significant differences in the FA (left) and MK (right) values in the various brain regions of controls and patients (NML or WL) at or below 3 years of age (*P < 0.05, **P < 0.01).

Two DKI parameters, FA and MK, were measured bilaterally in 12 brain regions (Fig. 2), including the auditory pathway and related cortex (trapezoid body, superior olivary nucleus, medial geniculate body, auditory radiation, and Heschl’s gyrus), language-related cortex (middle and inferior frontal gyri, middle and superior temporal gyri, angular gyrus, and supramarginal gyrus), and learning and memory function-related regions (hippocampus). Patients over 3 years of age had significantly lower FA and MK values in 6 white matter regions. However, in 2 other white matter regions of interest (ROIs) and 2 grey matter regions, only MK was lower, with no appreciable change in FA. In contrast, the younger patients (1 to 3-year-olds) had lower FA values in 3 white matter regions but no appreciable change in MK (Table 2). Figure 3 shows that compared with the healthy controls, patients older than 3 years with brain parenchymal lesions exhibited significant differences in terms of both FA and MK in five ROIs, whereas patients with normal MRI scans exhibited significant differences in both FA and MK in only 1 ROI (the superior temporal gyrus). More regions were detected with a lower MK or FA in patients with white matter lesions than in patients with undetectable lesions. Figure 4 shows that the patients under 3 years of age with brain parenchymal lesions had significant differences only in the FA of 4 ROIs (auditory radiation, Medial geniculate body, Heschl’s gyrus, and angular gyrus) and no appreciable change in MK.

**Table 2:** Comparison of FA and MK Values in the Various Brain Regions of Controls and Patients*.

	Children > 3 Years			Children 1–3 Years	
	Controls (*n* = 20)		SNHL (*n* = 40)	Controls (*n* = 18)	SNHL (*n* = 32)
	Mean ± SE		Mean ± SE	Mean ± SE	Mean ± SE
Auditory radiation	FA	0.495 ± 0.006	0.444 ± 0.005^‡^	0.466 ± 0.010	0.412 ± 0.007^‡^
	MK	0.869 ± 0.011	0.834 ± 0.011^†^	0.783 ± 0.012	0.790 ± 0.010
Trapezoid body	FA	0.491 ± 0.010	0.472 ± 0.007	0.439 ± 0.004	0.440 ± 0.007
	MK	1.031 ± 0.008	0.970 ± 0.015^†^	0.960 ± 0.018	0.925 ± 0.013
Superior olivary nucleus	FA	0.366 ± 0.008	0.354 ± 0.007	0.360 ± 0.011	0.333 ± 0.007
	MK	0.992 ± 0.015	0.929 ± 0.017^‡^	0.882 ± 0.018	0.886 ± 0.016
Medial geniculate body	FA	0.400 ± 0.008	0.392 ± 0.004	0.361 ± 0.014	0.371 ± 0.006
	MK	0.781 ± 0.018	0.738 ± 0.016	0.686 ± 0.023	0.676 ± 0.015
Heschl’s gyrus	FA	0.494 ± 0.007	0.316 ± 0.005^‡^	0.336 ± 0.009	0.298 ± 0.005^‡^
	MK	0.870 ± 0.012	0.834 ± 0.011^†^	0.561 ± 0.015	0.583 ± 0.009
Middle frontal gyri	FA	0.380 ± 0.007	0.351 ± 0.007^†^	0.362 ± 0.013	0.321 ± 0.007^†^
	MK	0.770 ± 0.018	0.703 ± 0.016^†^	0.599 ± 0.014	0.612 ± 0.0100
Inferior frontal gyri	FA	0.393 ± 0.010	0.357 ± 0.007^‡^	0.361 ± 0.013	0.341 ± 0.006
	MK	0.767 ± 0.021	0.724 ± 0.012^†^	0.619 ± 0.019	0.645 ± 0.009
Middle temporal gyri	FA	0.340 ± 0.010	0.351 ± 0.006	0.352 ± 0.009	0.341 ± 0.007
	MK	0.738 ± 0.021	0.688 ± 0.013^†^	0.579 ± 0.018	0.559 ± 0.021
Superior temporal gyri	FA	0.751 ± 0.066	0.431 ± 0.036^‡^	0.633 ± .0770	0.623 ± 0.072
	MK	0.466 ± 0.035	0.552 ± 0.020^‡^	0.393 ± 0.041	0.393 ± 0.041
Angular gyrus	FA	0.362 ± 0.013	0.325 ± 0.008^‡^	0.385 ± 0.043	0.385 ± 0.043
	MK	0.804 ± 0.026	0.698 ± 0.012^‡^	0.635 ± 0.020	0.635 ± 0.020
Supramarginal gyrus	FA	0.340 ± 0.009	0.332 ± 0.0061	0.331 ± 0.014	0.331 ± 0.014
	MK	0.743 ± 0.017	0.689 ± 0.012^†^	0.638 ± 0.018	0.638 ± 0.018
Hippocampus	FA	0.336 ± 0.010	0.317 ± 0.007	0.314 ± 0.012	0.314 ± 0.012
	MK	0.603 ± 0.015	0.580 ± 0.012	0.529 ± 0.025	0.529 ± 0.025

^*^The table demonstrates differences with *P* values of <0.01or <0.05 compared with the controls. Values are presented as the mean ± SD.

^†^Statistically significant difference: ^†^ *P* < 0.05, ^‡^ *P* < 0.01.

## Discussion

### DKI of microstructural alterations in the brain maturation of paediatric patients with SNHL

DKI has been used to resolve the crossing of white matter fibres and possibly lead to more accurate tracking and characterization. Recent experience has demonstrated the benefits of MK because it detects significant microstructural changes that are consistent with known patterns of brain maturation^24–28^. We hypothesized that DKI is a valuable technique for offering a more effective and comprehensive evaluation of age-related microstructural changes in both white and grey matter when they appear in various pathological states in SNHL patients, not only in the auditory region but also in the speech, learning and memory function–related regions, which may influence the outcome of CI. Kileny *et al*. demonstrated that the time of CI plays an important role in the development of auditory cortical myelination in children. SNHL children implanted between the ages of 12 and 36 months outperformed children implanted between the ages of 37 and 60 months^1, 26^. Patients in our study were divided into 2 groups according to age (children above 3 years of age, and children 1 to 3 years of age). The results demonstrated that the lack of normal stimulation by a variety of sounds leads to delayed developmental changes in both white and grey matter not only in the central auditory pathway but also in the language and cognitive-related cortex in children with SNHL and that this delay is correlated with the patient’s age. In contrast, the younger patients exhibited significant changes in FA but no appreciable change in MK in any region, which suggests that the maturation of crossing fibres in white matter is not markedly different between SNHL and normal-hearing children. Because more diffusion restriction occurs during brain maturation in both white and grey matter structures with age, the MK metric, as a new imaging tool, may better characterize the varying severity of those delayed developmental changes in patients with SNHL.

Moreover, in some grey regions in older SNHL children (language-related cortex and auditory pathway), there were significant changes in MK but no appreciable changes in FA in the same areas. These results demonstrate that DKI is sensitive to micro-structural changes that occur in isotropic grey matter (superior olivary nucleus), for which DTI has previously been demonstrated to exhibit limited sensitivity.

### Abnormal white matter microstructure in children with congenital sensorineural hearing loss

The analysis of our data revealed that 54.2% of paediatric CI recipients had a variety of abnormal findings in preoperative brain MR images, and there were no differences between different age groups. Several studies have demonstrated that children who exhibit preoperative structural CNS abnormalities may have poor postoperative outcomes and rehabilitation following CI^25, 29, 30, 31^. However, some paediatric candidates for CI with structural CNS abnormalities do not exhibit severe neurological symptoms. A substantial proportion of children with brain lesions are free of severe impairment, especially at young ages^25, 32^. At 3, 6, and 12 months post-surgery, we evaluated the CAP scores of the 20 patients who had received CI. Although some of them exhibited white matter lesions, they also exhibited gradual progress in auditory perception and speech production following CI. It is not clear whether patients with white matter lesions should be preoperatively counselled regarding the poor prognosis, and the decision of whether a patient with white matter lesions is suitable for CI remains controversial. Thus, to determine the correlation between postoperative outcome and the white matter changes noted on preoperative MRI studies, we used DKI to investigate patients with white matter lesions and found that those patients with a decreased MK in auditory, speech, learning and memory function-related regions were only in the older age group, which might indicate that the developmental delay of the brain was distinct, adversely affecting the postoperative rehabilitation and outcomes in children undergoing CI. This result involved preoperative counselling regarding the poorer prognosis, and these patients were followed up with special attention^27, 30^. In contrast, in younger children with SNHL, we found that regardless of whether they had white matter lesions, there was no significant change in the MK values in their brains, thus suggesting that there are no significant micro structural alterations to these patients’ brains, and the further development of their brains will be positive after CI during the brain maturation stage. These results may explain why some paediatric candidates for CI with structural CNS abnormalities do not exhibit severe neurological symptoms.

### Limitations

It should be noted that the current study has some limitations. First, the patient population was relatively small. Further studies with larger numbers of subjects are necessary. Second, the abnormalities detected on the whole-brain images were heterogeneous and of wide-ranging clinical significance. These brain lesions included white and grey matter lesions of varying severity. Due to the small number of patients with SNHL, we considered only subjects with white matter lesions (i.e., no grey matter lesions) for the DKI procedure. Third, as follow-up data become available, the correlation between the presence of abnormalities in FA or MK values from the auditory, language, and cognition areas of the brain and clinical outcomes (CAP, open sentence, open word, and closed set) will be investigated to provide further insight into their true significance. Finally, to the best of our knowledge, the conventional DKI acquisition time is relatively long, which limits its use in some clinical applications. Recently, the fast DKI approach has provided mean diffusion and kurtosis measurements with a substantially reduced scan time, thus making it amenable to acute imaging. Future fast DKI would be well suited for studies involving children^33–38^.

## Conclusions

DKI offers sensitive and comprehensive measurements for the quantitative evaluation of age-related micro structural changes in both white and grey matter in SNHL patients. It is therefore a valuable technique for detecting delayed development in the auditory, speech, and emotional-response regions of the brain in these patients. DKI scans in children with SNHL that exhibit significant decreases in MK might play an important role in evaluating the severity of developmental delay and judging the prognosis of CI.

## Methods

### Study Subjects

Seventy-two profound bilateral sensorineural hearing loss patients (38 males and 34 females between 1 and 7 years of age; mean age 4.7 ± 1.0 years) and 38 healthy controls (21 males and 17 females 1 to 8 years of age; mean age 4.2 ± 0.5 years) were recruited for this study. The inclusion criteria for the control group were (1) no previous history of neurological disorders or brain injury; (2) normal hearing, vision, and movement; and (3) no morphological abnormalities on conventional MRI. The children were all sedated with oral chloral hydrate (25–50 mg/kg) before scanning.

The hearing level was measured in a sound-proof booth using a calibrated pure tone audiometer (*Audiometer Grason*-*Stadler* GSI 61, USA). All the patients had profound bilateral sensorineural hearing loss prelingually (greater than 90 dB: averaged threshold 500 Hz, 1 and 2 kHz; pure tone average, PTA), and all used hearing aids before the imaging evaluation. Before the DTI examination, patients with anatomical abnormalities of the inner ear (cochlear aplasia, common cavity deformity, cochlear hypoplasia, or Mondini deformity) or cochlear nerve deficiency, as determined via CT and conventional MRI, were excluded. Of the 72 patients, 20 received CIs, and their CAP scores were evaluated at 3, 6, and 12 months postsurgery. The study protocol was designed in accordance with guidelines outlined in the Declaration of Helsinki and was approved by the Research Ethics Committee of the Second Affiliated Hospital, Medical College of Shantou University, Shantou, Guangdong, China. All of the participants provided written informed consent before each examination.

### MRI acquisition images

MRI was performed on all subjects using a 3.0-T GE MRI system (Signa; General Electric Medical System, USA) with an eight-channel head coil (GE Medical Systems). All patients underwent cranial conventional MRI to determine whether there were anatomical abnormalities of the inner ear or brain before the DKI scan. The conventional MR series included T1-weighted imaging (T1WI), T2-weighted imaging (T2WI), and fluid-attenuated inversion recovery (FLAIR). The DKI scans were performed with the following parameters: 6000/109.4 ms TR/TE; 4 mm slice thickness; 24 sections; 240 × 240 mm field of view; 256 × 256 matrix; average = 1; 4 minutes and 10 seconds imaging time; and 3b values (0, 1000, 2000 s/mm^2^), with diffusion encoding in 15 directions for every value. The first images are the b0 images, which were coregistered by the preprocess_options.extra_b0 function of DKE.

### Data analysis

All images were obtained and transferred to a workstation (Advantage Workstation 4.6; GE Medical Systems), and the Functool software package was used for data processing. The DKI software is a research tool in the Functool environment developed by GE Applied Science Lab (see http://www.nitrc.org/projects/dke/). It fits all diffusion-weighted images (DWIs) and the minimally diffusion-weighted image (b_0_ image) to the DKI model described by Equation (1),

1 $$\mathrm{ln}\,[S(n,b)/{S}_{0}]=-\,b\sum _{i=1}^{3}\sum _{j=1}^{3}{n}_{i}{n}_{j}{D}_{ij}+\frac{1}{6}{b}^{2}{\bar{D}}^{2}\sum _{i=1}^{3}\sum _{j=1}^{3}\sum _{k=1}^{3}\sum _{l=1}^{3}{n}_{i}{n}_{j}{n}_{k}{n}_{l}{W}_{ijkl},$$

where S(n, b) is the diffusion signal intensity for diffusion weighting b and diffusion encoding direction **n**, S_0_ is the signal intensity for b_0_, and D_ij_ and W_ijkl_ are the components of the diffusion and kurtosis tensors, respectively. After the tensors were estimated, and the DKI parameters of FA, MD, Da, Dr, FA, MK, Ka and Kr and the DTI parameters of FA, MD, Da, and Dr were derived. The DKI analysis steps described above were also used in a study by Wang M.Y. *et al*.^39^.

The ROI was approximately 14 mm^2^ and was traced on the auditory pathway, auditory cortex, language-related cortex, and learning and memory function–related cortex in the original DKI. Two DKI parameters, FA and MK, were measured bilaterally in 12 brain regions (Fig. 2), including the auditory pathway and related cortex (trapezoid body, superior olivary nucleus, medial geniculate body, auditory radiation, and Heschl’s gyrus), language-related cortex (middle and inferior frontal gyri, middle and superior temporal gyri, angular gyrus, and supramarginal gyrus), and learning and memory function–related regions (hippocampus). The FA and MK values for the ROIs were acquired and averaged over three repeats to improve the signal-to-noise ratio. The superior olivary nucleus was measured only once because its small size hampered accurate measurements. The average values of the data were measured by 2 experienced radiologists and analysed for structural abnormalities, including microinfarcts and gliotic spots in the region of the small penetrating arteries and arterioles. We measured the AK, RK, AD and RD, but they were excluded because the data were too noisy to analyse.

### Statistical analysis

All analyses were performed with SPSS 20.0 for Windows (SPSS, Chicago, IL, USA). The chi-square test was used to compare the proportions of patients with abnormalities detected in the whole-brain images in the 2 age groups. Each of the measured parameters (MD, FA, and MK) was expressed as the mean ± standard deviation (SD). A one-way analysis of variance (ANOVA) was performed to compare groups. If the overall test of the two means for the FA and MK values showed statistically significant differences, then multiple comparisons of the means of any two groups were made by using the least significant difference (LSD). Two levels of statistical significance, *P* < 0.05 and *P* < 0.01, were considered.
